# 41. RECONSIDERING THE EVIDENCE FOR CLOZAPINE FOR TREATMENT REFRACTORY SCHIZOPHRENIA

**DOI:** 10.1093/schbul/sby014.168

**Published:** 2018-04-01

**Authors:** James MacCabe

**Affiliations:** Institute of Psychiatry, Psychology and Neuroscience, King’s College London

## Abstract

**Overall Abstract:** The superiority of clozapine for treatment refractory schizophrenia was, until very recently, seen as one of the few unshakeable truths in psychiatry. But the pre-eminence of clozapine has recently been called into question by meta-analyses. What are we to make of the fact that meta-analyses of clinical trials, supposedly the pinnacle of evidence based medicine, fail to show an effect which seems clearly evident to most clinicians, and on which many of our guidelines are based? Have we believed in a fairytale for the past three decades? Or do biases in RCTs and methodological limitations of meta-analyses explain the results? These questions will be discussed by Dan Siskind.

Another way to address the question of efficacy is to examine the pharmaco-epidemiological evidence using population-based registers. Jari Tiihonen will present data from his seminal studies of mortality and readmission rates under clozapine treatment versus other antipsychotics, as well as other data. These data seem to show powerful positive effects of clozapine at the population level. Furthermore, more recent evidence suggests a role for clozapine in reducing rates of violent offending, with new data presented for the first time by Vishal Bhavsar.

Finally, despite clinical guidelines recommending the use of clozapine, the actual rates of clozapine use are much lower than expected, with large regional and international variations. There is evidence that the burden of blood monitoring deters physicians from prescribing clozapine. Yvonne van der Zalm will present new data from a cluster randomised trial testing the efficacy and safety of an intervention to increase rates of clozapine prescribing by employing nurse practitioners trained in the initiation and monitoring of clozapine.

John Kane, author of the first, seminal RCT of clozapine in 1988, will lead the discussion.

